# Supplementary material for: Genome Analysis of a Variant of Streptomyces coelicolor M145 with High Lipid Content and Poor Ability to Synthetize Antibiotics
Source: Microorganisms. 2023 May 31;11(6):1470. doi: 10.3390/microorganisms11061470 (PMC10302056; doi:10.3390/microorganisms11061470)
Supplement: Supplementary file 1 [file microorganisms-11-01470-s001.zip › microorganisms-2396096-supplementary.pdf]

**Supplementary Table S1**

**Oligonucleotides**

Amplification of the regions located in 5′ and 3′ of *sco0982*

5′_*sco0982*_forward: GGCAAGCTTCGTTCCAGAGCGGGCAGGTG *Hind*III

5′_*sco0982*_reverse: TTTGATATCGCTCGATGCCCTGCCACCTC *Eco*RV

3′_*sco0982*_forward : AAAGATATCACCAAATCGCCAAGTTCCAGCGC *Eco*RV

3′_*sco0982*_reverse: TAAGGATCCGGAGGAGGAAGTCGGTGCGGTG *Bam*HI

Verification of the replacement of *sco0982* by the *aac(3)IV* cassette

5'_*sco0982*_verif_forward: GGCATCTCGGACAGCTGG

5′_*aac(3)IV*_verif_reverse : GGATTTGAACCCACGACCTC

3′_*aac(3)IV*_ verif_forward: ACGACATTGCACTCCACCGC

3'_*sco0982*_verif_reverse: CGATGTTGGTGCGCAGGC

Amplification of *sco0984* coding sequence

*sco0984*_forward GAGAAGCTTGAGAAGGAGCGGACATGAGCGGGGCATCCGTC *Hind*III

*sco0984*_reverse CTTCTGCAGTTCAGTCCCGGTCGTAGGTGTG *Pst*I.

Figure S1

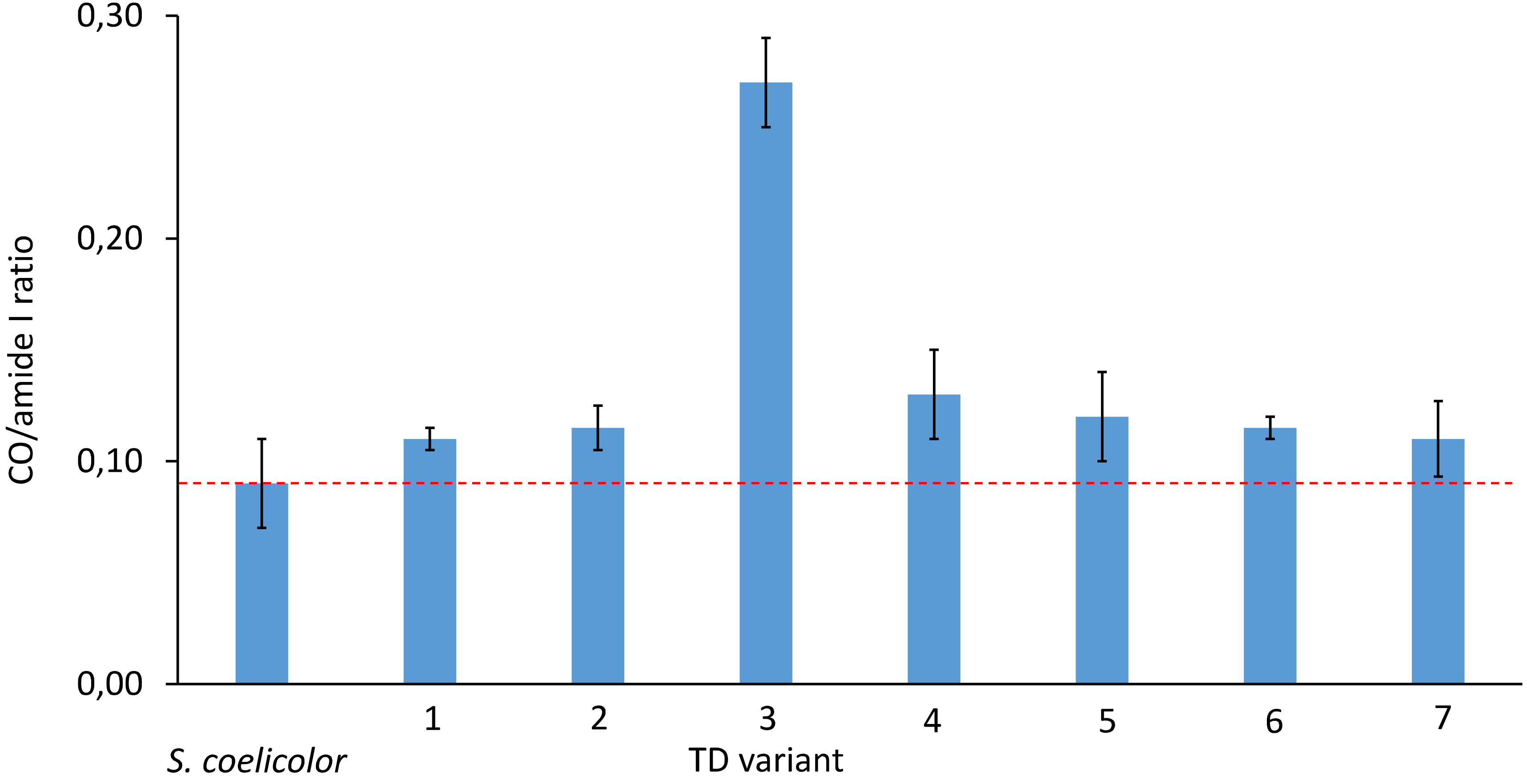

0,30
0,20
0,10
0,00
CO/amide I ratio
1
2
3
4
5
6
7
S. coelicolor
TD variant

Figure S2

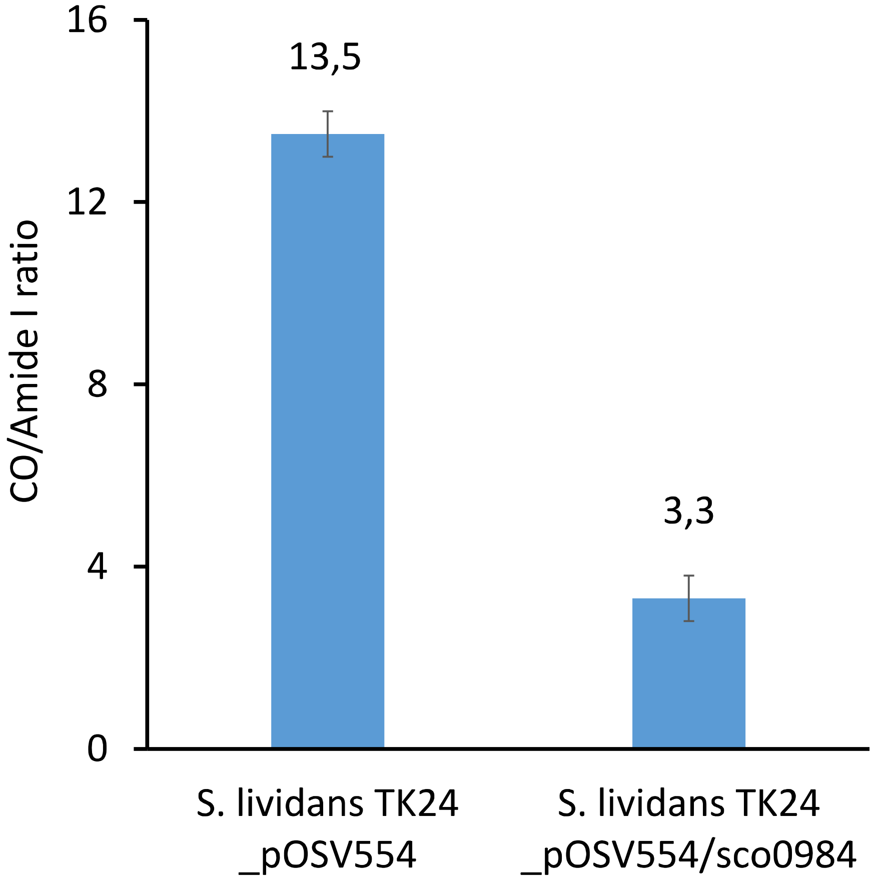

16
12
8
4
0
CO/Amide I ratio
13,5
3,3
S. lividans TK24 _pOSV554
S. lividans TK24 _pOSV554/sco0984
